# Substrate metabolism in male astronauts onboard the International Space Station: the ENERGY study

**DOI:** 10.1038/s41526-024-00360-0

**Published:** 2024-03-27

**Authors:** Elisa Le Roux, Alexandre Zahariev, Isabelle Chery, Dale A. Schoeller, Pierre Bourdier, Alain Maillet, Cecile Thevenot, Maël Garnotel, Guillemette Gauquelin-Koch, Laurie Van Den Berghe, Stéphane Blanc, Chantal Simon, Audrey Bergouignan

**Affiliations:** 1 Université de Strasbourg, CNRS, IPHC UMR 7178, Strasbourg, F-67000 France; 2 Department of Nutritional Sciences, University of Wisconsin, Madison, WI USA; 3 MEDES, Institut de Médecine et Physiologie Spatiale, Toulouse, France; 4 CADMOS-CNES, Toulouse, France; 5 Centre National d’Etudes Spatiales, Paris, France; 6 Human Nutrition Research Centre of Rhône-Alpes, Hospices Civils de Lyon, Lyon, France; 7 CarMen Laboratory, INSERM 1060, INRA 1397, University of Lyon, Oullins, France; 8 Division of Endocrinology, Metabolism and Diabetes, Anschutz Health & Wellness Center, Anschutz Medical Campus, University of Colorado, Aurora, CO USA

**Keywords:** Physiology, Biological techniques

## Abstract

Bedrest shifts fasting and postprandial fuel selection towards carbohydrate use over lipids, potentially affecting astronauts’ performance and health. We investigated whether this change occurs in astronauts after at least 3 months onboard the International Space Station (ISS). We further explored the associations with diet, physical activity (PA), and body composition. Before and during spaceflight, respiratory quotient (RQ), carbohydrate, and fat oxidation were measured by indirect calorimetry before and following a standardized meal in 11 males (age = 45.7 [SD 7.7] years, BMI = 24.3 [2.1] kg m^−^²). Postprandial substrate use was determined by 0-to-260 min postprandial incremental area under the curve (iAUC) of nutrient oxidation and the difference between maximal postprandial and fasting RQ (ΔRQ). Food quotient (FQ) was calculated from diet logs. Fat (FM) and fat-free mass (FFM) were measured by hydrometry and PA by accelerometry and diary logs. Spaceflight increased fasting RQ (*P* = 0.01) and carbohydrate oxidation (*P* = 0.04) and decreased fasting lipid oxidation (*P* < 0.01). An increase in FQ (*P* < 0.001) indicated dietary modifications onboard the ISS. Spaceflight-induced RQ changes adjusted for ground RQ correlated with inflight FQ (*P* < 0.01). In postprandial conditions, nutrient oxidation and ΔRQ were unaffected on average. Lipid oxidation changes negatively correlated with FFM changes and inflight aerobic exercise and positively with FM changes. The opposite was observed for carbohydrate oxidation. ΔRQ changes were negatively and positively related to FM and FFM changes, respectively. In conclusion, fasting substrate oxidation shift observed during spaceflight may primarily result from dietary modifications. Between-astronaut variability in postprandial substrate oxidation depends on body composition changes and inflight PA.

## Introduction

Microgravity leads to numerous physiological alterations, including dysfunctions of metabolism^1,2^. Because metabolism is at the crossroads of multiple physiological functions^3,4^ such as cardiovascular and musculoskeletal functions, it can affect astronauts’ performance and health. However, the consequences of several months of spaceflight on nutrient metabolism are poorly known.

Space model analogs on Earth, i.e., the head down tilt bedrest^5^, showed in healthy male and female adults that 7, 42, 60, and up to 90 days of exposure to simulated microgravity induces a shift in substrate utilization characterized by a decrease in fat oxidation in favor of the use of carbohydrates as fuel^6–9^. This was observed in both fasting and postprandial states, and so far, it has been seen as independent of changes in body composition and diet. Bedrest studies also showed that microgravity reduces the shift from fat oxidation in the fasting state to the preferential use of carbohydrates as fuel following meal consumption^10^. In a 21-day bedrest study, we also showed that the latter preceded the onset of glucose intolerance in the pathophysiological context of insulin resistance in healthy men^11^. This change in substrate oxidation, with other metabolic adaptations induced by microgravity, is part of the metabolic alterations observed in people with chronic metabolic pathologies such as type 2 diabetes, obesity, or metabolic syndrome^2^. It is, therefore, important to determine whether a change in substrate oxidation develops in space, which has never been done before.

Changes in substrate oxidation in response to actual microgravity in space may differ from what was observed under simulated microgravity conditions using space model analogs on Earth. Indeed, all astronauts are asked to observe an exercise training protocol aiming to mitigate the microgravity-induced body alterations^12^, and no true control (no exercise) group exists in space. This being said, a combined aerobic and resistance exercise training protocol similar to the one applied on the International Space Station (ISS) only partially prevented the shift in substrate oxidation towards carbohydrate use in both fasting and postprandial states during a 2-month bedrest study^8^. It is also important to note that a wide between-subject variability in body composition and energy metabolism in response to a 3-to-5-month spaceflight was recently reported in the ENERGY experiment^13^, with ground physical activity and inflight exercise being major factors in this variability. Similar inter-individual variations may exist in the nutrient metabolism response.

In addition to investigating the relationships of energy expenditure, energy intake, and body composition with the physical exercise countermeasure^13^, the ENERGY experiment included a one-day metabolic test combining fasting and post-meal responses in substrate oxidation. This study aimed to determine whether a shift in substrate oxidation developed in space, both in the fasting state and after a mixed meal, as reported during bedrest studies. We also used the wide between-subject variability observed in the ENERGY experiment to examine the associations with diet, physical activity, and body composition.

## Results

### Subjects’ ground characteristics

At baseline (Table 1), astronauts had a mean age of 45.7 years (SD 7.7), were normal-weight with a mean BMI of 24.3 kg.m^−2^ (SD 2.1), and presented a normal-to-high FFMI (19.6 kg m^−2^ [SD 1.9]). The latter was consistent with a fairly high daily time spent physically active; the total SenseWear Pro (SWP)-derived physical active time was estimated at 162 min.d^−1^ (SD 56), and included 10,077 daily steps (SD 2834), 67 min.d^−1^ walking (SD 34) and 8 min.d^−1^ running (SD 10).

**Table 1:** Ground and inflight body composition and physical activity characteristics of the 11 astronauts

	Ground	Inflight	Changes from ground^1^	
	Mean (SD)	Mean (SD)	Lsmeans (95% CI)	*P* value
Anthropometry and body composition				
Body mass (kg)	79.4 (10.6)	78.2 (11.0)	−1.2 (−2.3 to −0.1)	0.04
Body mass index (kg.m^−2^)	24.3 (2.1)	23.9 (2.1)	−0.4 (−0.8 to −0.0)	0.04
Fat-free mass (kg)	63.8 (8.8)	62.8 (9.0)	−0.9 (−2.3 to +0.4)	0.14
Fat-free mass index (kg.m^−2^)	19.6 (1.9)	19.3 (2.1)	−0.3 (−0.7 to +0.1)	0.13
Fat mass (kg)	15.6 (3.9)	15.3 (5.5)	−0.3 (−2.2 to +1.7)	0.77
Fat-free mass index (kg.m^−2^)	4.8 (1.1)	4.7 (1.5)	−0.1 (−0.7 to +0.5)	0.70
Physical activity				
SWP-derived^2^				
Total activity & exercise (min.day^−1^)	162 (56)	175 (48)	13.2 (−36.8 to +63.2)	0.56
Walking (min.day^−1^)	66.7 (34.2)	3.6 (2.6)	−63.2 (−87.3 to −39.1)	<0.001
Running (min.day^−1^)	8.11 (10.49)	14.4 (6.4)	6.3 (−2.1 to +14.7)	0.13
Inflight training				
Aerobic exercise (min.week^−1^)		168 (44)		.
Resistive exercise (rep.week^−1^)		1481 (834)		.

*SWP* Sensewear Pro activity device; aerobic exercise, T2 + CEVIS; resistive exercise, ARED.

^1^Estimated LSmeans (95% CI) and *P* value from mixed-effects models accounting for repeated values. ^2^Inflight data from nine subjects.

### Changes in body composition and physical activity during flight

Compared to preflight ground values (Table 1), after at least three months onboard the ISS, body mass slightly decreased by 1.20 kg (95% CI −2.3 to −0.1, *P* = 0.04) mainly due to a non-significant reduction in fat-free mass (FFM) of 0.9 kg (95% CI −2.3 to 0.4, *P* = 0.14). However, these numbers masked a wide inter-individual variability with changes in FFM ranging from −5.4 kg to + 1.3 kg and fat mass (FM) from −3.5 kg to +5.3 kg.

As expected, spaceflight was associated with a dramatic drop in SWP-derived walking time, which represented only less than 4 min.d^−1^ inflight. Mean daily SWP-derived overall physically active time was not significantly modified, partly due to the exercise countermeasure program (Table 1). The astronauts reported exercising for a total of 168 min.week^−1^ (SD 44) on either the cycle ergonometer (CEVIS) or the second-generation treadmill (T2), and 4.5 resistance training sessions.week^−1^ (SD 1.8) on the advanced resistive exercise device (ARED) leading to a total of 1481 repetitions.week^−1^ (SD 834). As for anthropometric data, a high inter-individual variability in the adherence to the exercise prescription was noted, with time spent in aerobic exercise ranging from 87 to 258 min.week^−1^, and the number of repetitions on the ARED ranging from 468 to 3044 per week. More information on physical activity was recently published^13^.

### Three to five months of spaceflight is associated with a metabolic shift towards carbohydrate oxidation, mainly due to changes in food substrate availability

**Fig. 1:**
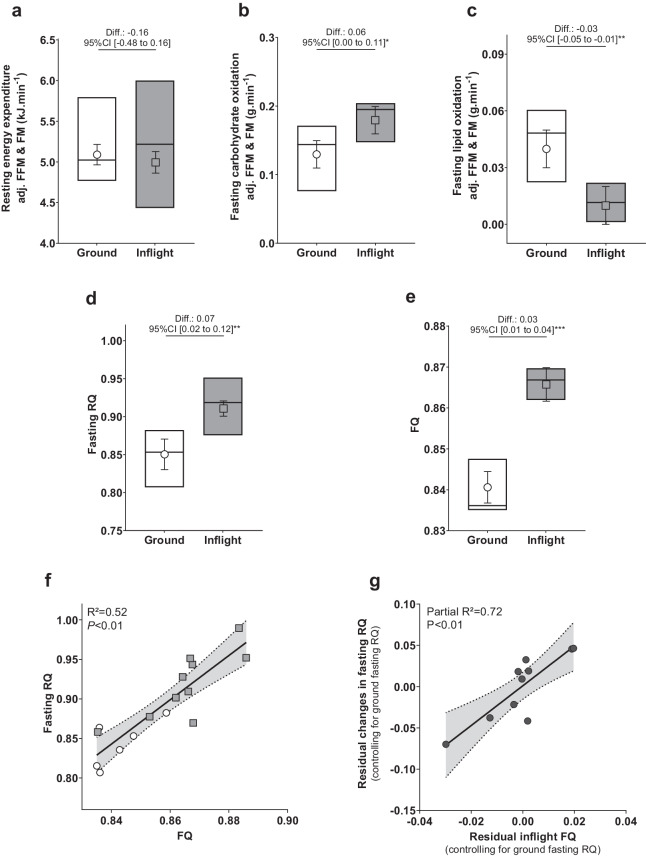
Fasting resting energy expenditure, substrate oxidative rates, respiratory quotient, and food quotient on the ground and inflight. Ground and inflight fasting resting energy expenditure (**a**), carbohydrate oxidation rate (**b**), lipid oxidation rate (**c**), respiratory quotient (RQ, **d**), and food quotient (FQ, **e**). Values are interquartile range with median and lsmeans (SE) from mixed-effects models accounting for repeated measurements, with differences (Diff.) presented with their 95% confidence interval. Models for energy expenditure and substrate oxidation rates are adjusted for FFM and FM. *P < 0.05, **P < 0.01, ***P < 0.001. Scatterplot of individual ground (white circles) and inflight (gray squares) fasting RQ with corresponding FQ (**f**). Illustration of the partial relationship of changes in fasting RQ (difference between inflight fasting RQ and ground fasting RQ) with inflight FQ adjusted for ground RQ using the scatterplot of the relationships of the residual changes in fasting RQ (controlling for ground RQ) with the residual inflight FQ (controlling for ground fasting RQ; **g**). Least square regression lines are plotted with their 95% confidence interval (gray area). **a**–**d**, **f**: inflight data from 10 subjects; e: ground data from 7 subjects; **g** data from 10 subjects. FFM fat-free mass, FM fat mass, RQ respiratory quotient, FQ food quotient.

Values of energy expenditure and absolute substrate oxidation rates controlled for FM and FFM are presented (data from unadjusted models are presented in Supplemental Table 2). As illustrated in Fig. 1 and detailed in Table 2, three months in space did not significantly alter fasting energy expenditure compared to ground values (Fig. 1a). However, it was associated with a pronounced increase in carbohydrate oxidation rate (+0.06 g.min^−1^ [95% CI 0.00–0.11], *P* = 0.04; Fig. 1b) along with a reduction in fasting fat oxidation rate (−0.03 g.min^−1^ [95% CI −0.05 to −0.01], *P* < 0.01; Fig. 1c). Consequently, fasting RQ significantly increased by 0.07 (95% CI 0.02–0.12, *P* < 0.01; Fig. 1d). Carbohydrates represented 65% (SE 5) of fasting energy expenditure inflight as compared to 44% (SE 5) on the ground (*P* = 0.01), while the contribution of fat oxidation to fasting energy expenditure decreased from 34% (SE 6) to 10% (SE 3) (*P* < 0.01).

**Table 2:** Ground and inflight nutrition and substrate oxidation data of the 11 astronauts

	Ground	Inflight	Changes from ground^1^	
	LSmeans (SE)	LSmeans (SE)	LSmeans (95% CI)	*P* value
Fasting state^3^				
Energy expenditure (kJ.min^−1^)^2^	4.73 (0.20)	4.58 (0.21)	−0.16 (−0.48 to 0.16)	0.30
Carbohydrate oxidation (g.min^−1^)^2^	0.13 (0.02)	0.18 (0.02)	0.06 (0.00–0.11)	0.04
Lipid oxidation (g min^−1^)^2^	0.04 (0.01)	0.01 (0.01)	−0.03 (−0.05 to −0.01)	<0.01
Respiratory quotient (RQ)	0.85 (0.02)	0.91 (0.02)	0.07 (0.02–0.12)	<0.01
Carbohydrate oxidation (%)	44 (5)	65 (5)	21 (6–36)	0.01
Lipid oxidation (%)	34 (6)	10 (3)	−23 (−37 to −9)	<0.01
Protein oxidation (%)	22 (1)	24 (1)	2 (0–4)	0.02
Post-breakfast challenge (iAUC over 260 min)^3^				
Energy expenditure iAUC (kJ)^2^	302 (29)	452 (45)	150 (36–264)	0.01
Carbohydrate oxidation iAUC (g)^2^	16.8 (6.4)	22.3 (2.5)	5.5 (−9.5 to 20.5)	0.44
Lipid oxidation iAUC (g)^2^	0.1 (2.3)	3.1 (0.8)	2.1 (−3.3 to 7.5)	0.41
ΔRQ	0.06 (0.02)	0.05 (0.02)	−0.02 (−0.07 to 0.04)	0.56
Diet^4^				
Food quotient (FQ)	0.84 (0.00)	0.87 (0.00)	0.03 (0.01 to 0.04)	<0.001

*iAUC* incremental area under the curve over the 260 min period of measurement, *FFM* fat-free mass, *FM* fat mass, ΔRQ maximal postprandial RQ–fasting RQ

^1^Estimated LSmeans (95% CI) and *P* value from mixed-effects models accounting for repeated values.

^2^Models for energy expenditure and absolute carbohydrate and lipid oxidation rates are adjusted for FFM and FM.

^3^Inflight data from 10 subjects

^4^Ground data from 7 subjects.

Concomitantly to this fasting metabolic shift, changes in diet composition were observed with an increase in the proportion of carbohydrates in the diet energy intake at the expense of fat contribution. These dietary changes translated into a mean inflight FQ of 0.87 (SD 0.01) as compared to an FQ of 0.84 (SD 0.01) obtained in 7 subjects on the ground (*P* < 0.001; Table 2 and Fig. 1e). Individual inflight and ground RQ were significantly associated with corresponding FQ (*R*^2^ = 0.52; *P* < 0.01; Fig. 1f). Mediating analyses indicated that FQ explained 69% of the changes in RQ induced by spaceflight (*P* < 0.01). Multiple regression analysis indicated that changes in fasting RQ adjusted for ground RQ were significantly associated with inflight FQ (partial *R*^2^ = 0.72; *P* < 0.01; Fig. 1g); inflight FQ and ground RQ explaining together 90% of the variance observed in the spaceflight-induced RQ changes (Supplemental Table 3).

The results of the fasting metabolic shift did not correlate with any anthropometric or physical activity measurements. Of note, there was also no correlation with either the time interval between the ground and inflight sessions or the time spent in space (data not shown).

### Spaceflight does not alter postprandial substrate oxidation in response to a breakfast challenge

**Fig. 2:**
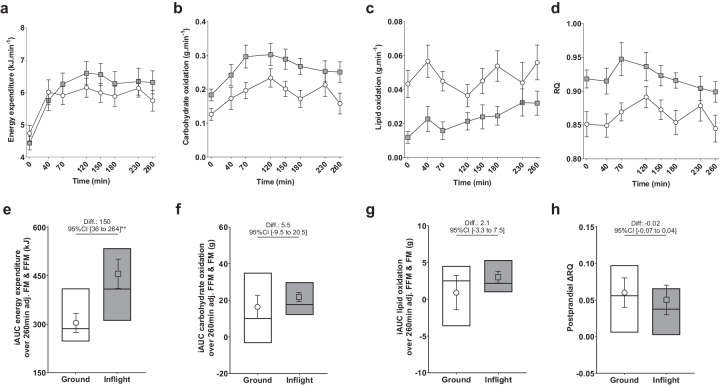
Response of energy expenditure, substrate oxidation, and respiratory quotient to the breakfast challenge on the ground and inflight. Kinetics and corresponding iAUC over the 260-min postprandial period of the breakfast-meal challenge of energy expenditure (**a**, **e**), carbohydrate oxidation (**b**, **f**), and lipid oxidation (**c**, **g**) on the ground and inflight. Kinetics of RQ during the breakfast-meal challenge (**d**) and ΔRQ (**h**), used as an index postprandial change in substrate use, on the ground and inflight. Kinetics are presented by ground (white circles) and inflight (gray squares) unadjusted means (SE) over time. Incremental AUCs and ΔRQ are interquartile range with median and lsmeans (SE) from mixed-effects models accounting for repeated measurements with differences (Diff.) presented with their 95% confidence interval. Models for energy expenditure and substrate oxidation iAUC are adjusted for FFM and FM. *P < 0.05, **P < 0.01, ***P < 0.001. FFM fat-free mass, FM fat mass, RQ respiratory quotient, ΔRQ difference between maximal post meal RQ and fasting RQ; iAUC, incremental area under curve over the 260 min postprandial period. **a**–**d**, **f**–**h**: inflight data from 10 subjects.

Figure 2a–d represents unadjusted kinetics for energy expenditure, carbohydrates, fat oxidation, and RQ after ingesting a breakfast challenge (time = 0–260 min) during the ground and inflight sessions. The test meal represented 62.3% (SD 10.1) and 67.8% (SD 10.6) of the measured resting energy expenditure on the ground and inflight, respectively. Post-breakfast incremental area under the curve (iAUC) of energy expenditure and carbohydrate and fat oxidation values, adjusted for FFM and FM, are presented in Fig. 2e–g and Table 2.

Post-breakfast energy expenditure iAUC adjusted for FFM and FM was higher inflight than on the ground by 150 kJ (95% CI 36–264, *P* = 0.01; Fig. 2e).

Metabolic responses to the breakfast challenge were characterized by lower inter-individual variability inflight than on the ground (Table 2; Fig. 2f, g) for both carbohydrate oxidation iAUC (interquartile range [IQR] 8.7 g inflight vs 20.1 on the ground) and fat oxidation iAUC (IQR 3.1 g inflight vs. 7.2 on the ground). However, spaceflight did not impact mean iAUC of carbohydrate oxidation (+5.5 g [95% CI −9.5 to 20.5], *P* = 0.44; Fig. 2f) and fat oxidation (+2.1 g [95% CI −3.3 to 7.5], *P* = 0.41; Fig. 2g), both being adjusted for FFM and FM. Mean RQ following ingestion of the breakfast challenge remained higher inflight than on the ground, which is in line with the metabolic shift observed toward the use of carbohydrates as fuel in the fasting state. The difference between maximal post-breakfast RQ and fasting RQ (ΔRQ) was not modified inflight compared to ground values (−0.02 [95% CI −0.07 to +0.04], *P* = 0.56; Fig. 2h).

### Associations of changes in post-breakfast substrate oxidation with changes in body composition and exercise

**Fig. 3:**
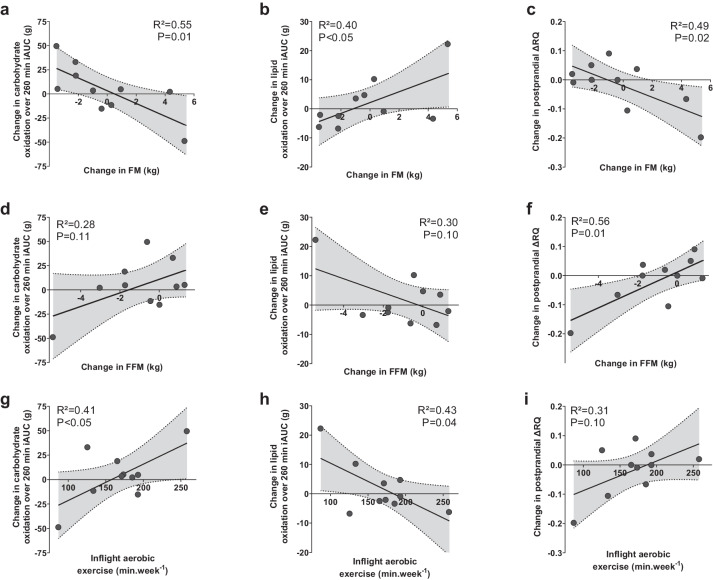
Associations between spaceflight-induced changes in post-breakfast substrate oxidation and both body composition changes and inflight exercise. Scatterplots for the relationships of spaceflight-induced changes in post-breakfast carbohydrate lipid oxidation incremental area under the curve (iAUC) and ΔRQ with FM changes (**a**–**c**, respectively), FFM changes (**d**–**f**, respectively) and self-reported inflight exercise (**g**–**i**, respectively). The “changes” term refers to the difference between inflight and ground values. Least square regression lines are plotted with their 95% confidence interval (gray area). iAUC incremental area under curve over the 260 min period, ΔRQ difference between maximal post meal RQ and fasting RQ. **a**–**d**, **f**–**i**: inflight data from 10 subjects.

Spaceflight-induced changes in the metabolic response to the breakfast challenge were associated with spaceflight-induced changes in FFM and FM and with inflight aerobic exercise (Fig. 3). FM changes were positively associated with changes in post-breakfast lipid oxidation iAUC (*R*^2^ = 0.40, *P* < 0.05; Fig. 3b) but negatively with changes in carbohydrate oxidation iAUC (*R*^2^ = 0.55, *P* = 0.01; Fig. 3a). Changes in ΔRQ were negatively and positively associated with changes in FM (*R*^2^ = 0.49, *P* = 0.02; Fig. 3c) and FFM (*R*² = 0.56, *P* = 0.01; Fig. 3f), respectively. Finally, changes in carbohydrate and lipid oxidation iAUC were positively (*R*² = 0.41, *P* < 0.05; Fig. 3g) and negatively (*R*² = 0.43, *P* = 0.04; Fig. 3h) associated with inflight time spent in aerobic exercise, respectively.

## Discussion

The ENERGY study aimed to investigate energy and nutrient metabolism in astronauts during a 6-month flight onboard the ISS. The effects of exposure to microgravity on energy expenditure and body composition during the ENERGY study were previously published^13^. The present study reported the effects on nutrient use estimated in the fasting state and in response to a standardized mixed meal. As it has been repeatedly reported in bedrest studies for the past 30 years, a shift in total substrate use in favor of carbohydrates and detriment to lipid oxidation was observed in space. However, dietary intake changes appeared to be the main factor in this shift. Contrary to what was observed in response to simulated microgravity on Earth, there was no decrease in postprandial ∆RQ. However, because of the wide inter-individual variability seen on the ground and, to a lesser extent, inflight, associations between changes in post-breakfast nutrient oxidation and both changes in body composition and inflight exercise performance could be found.

Four bedrest studies in healthy adults ranging in duration from 7 to 90 days have shown that simulated microgravity induces a shift in nutrient oxidation that favors the use of carbohydrates as fuel in fasting and fed states^6–9^. This shift was noticed under conditions of positive, stable, and negative energy balance^14^, suggesting that it was unrelated to energy balance. However, it was prevented by high volumes of exercise, at least partially^2,15^. For instance, a 60-day bedrest study reported that an average of 30 min.day^−1^ of moderate-to-vigorous intensity aerobic exercise combined with an average of 12 min/day of resistive exercise significantly mitigated the increase in fasted and postprandial non-protein respiratory quotient (NPRQ) induced by strict bedrest in healthy women adults^8^. In the present study, astronauts self-reported on average 24 min.day^−1^ of aerobic exercise using both the T2 and CEVIS devices and 1481 repetitions per week using the ARED device^13^, which is approximately equivalent to 29 min.day^−1^ of resistive exercise. Despite engaging in volumes of exercise roughly equivalent to those tested on the ground, the exercise countermeasure was insufficient to prevent the shift in nutrient use during the spaceflight. This may be because microgravity and/or physical inactivity are not the primary determinants of the shift in nutrient use. Using dietary surveys, we estimated a rough increase in carbohydrate intake from 43% of energy intake in the habitual diet of the astronauts on the ground to 50% of energy intake inflight. A concomitant approximate decrease in fat intake from 34% to 30% of energy intake was estimated. Recently, Péronnet and Haman extensively reviewed the relationship between intrinsic substrate oxidation, energy balance, FQ, and fasting RQ^16^. They discussed how fasting RQ intra- and inter-subject variabilities may be related to various factors that are difficult to control and not fully identified. Among these, fasting RQ is known to be influenced by energy balance and diet macronutrient composition^17,18^. In a study that aimed to determine the variability and determinants of resting RQ in trained cyclists, a multivariate analysis showed that 59% of the variance in fasting RQ was explained by type I muscle fiber content, muscle glycogen content, training volume, plasma free fatty acid and lactate concentration, and the percentage of dietary fat intake^19^. We observed that 90% of the variations observed in the changes in fasting RQ induced by the spaceflight were related to inflight nutritional intake along with baseline RQ. This observation suggests that individual oxidative capacity and changes between ground and inflight diets, primarily drive the spaceflight-induced shift in substrate use. Nevertheless, we cannot rule out the possibility that digestion and/or assimilation of nutrients may be affected by microgravity^20^, and that the dependence of fasting RQ on FQ may be different in space and on the ground. Muscle biopsies collected before and after the flight would have helped determine whether the structural, functional, and molecular adaptations widely described in studies of prolonged bedrest^2^ also participate in the variation of inflight fasting substrate oxidation, like what was observed in the trained cyclists^19^. Of note, decreases in type I muscle fibers have been repeatedly observed in animals spending between 11 and 91 days in space^21–26^ and in men and women bedrested from 55 to 84 days^27–30^. Without blood samples and muscle biopsies, we cannot exclude the role of muscle oxidative capacity alteration in response to microgravity. Together, these data suggest that inflight diet may predict the shift in substrate use observed during spaceflight more than microgravity per se.

In this study, we also assessed substrate use in response to a standardized, moderately high-fat and high-protein mixed meal that allowed the reproduction of the dynamic processes of absorption and digestion of complex foods and the use of mixed substrates. We showed that postprandial ∆RQ was unchanged after 3–5 months of exposure to microgravity. A decrease in postprandial ∆RQ would be significant as it favors the development of ectopic fat storage^8,31,32^. Lipid accumulation in skeletal muscle has been associated with systemic and muscle insulin resistance^33^ and muscle atrophy^34^, which are key physiological changes classically observed during bedrest studies^7–9,35–38^ as well as in astronauts after 4 days and 6 months in space^39–45^. Given that the changes in basal substrate oxidation are primarily explained by the dietary changes between the inflight diet, and habitual diet, one could question whether those dietary changes may have influenced the assessment of postprandial ∆RQ and/or the changes in postprandial ∆RQ. As far as we know, only one clinical research study has assessed the impact of diet on postprandial ∆RQ. By comparing the effect of two 6-week eucaloric diets (western vs. “healthy” diet) differing in carbohydrate (48% vs. 40% of energy intake) and protein (14% vs. 20% of energy intake) intakes^46^, the authors showed that both fasting RQ and RQ measured in response to a high-fat mixed-meal challenge (42% of carbohydrates, 51% of fat, 7% of protein) were lower in the group who consumed the “healthy” diet compared to those who consumed the western diet. However, the difference in postprandial RQ between the two groups did not persist after adjusting for differences in fasting RQ, which suggests no influence of the habitual diet on the postprandial use of substrates as fuel. In line with these data, we observed no relationship between changes in postprandial ∆RQ and inflight FQ (data not shown). Together, these data suggest that inflight diet largely contributes to the shift in fasting substrate oxidation but likely does not affect postprandial substrate use in astronauts, at least in response to a standard meal enriched in fat and proteins.

We showed that flight-induced changes in postprandial substrate use were associated with changes in body composition and exercise. Changes in FM were positively associated with changes in postprandial lipid oxidation, while postprandial carbohydrate oxidation and postprandial ∆RQ were negatively correlated. A recent systematic review^47^ that combined clinical studies on the relationship between adiposity and ∆RQ in response to metabolic challenges reported inverse correlations between ∆RQ to glucose plus insulin stimulation (i.e., measured during a hyperinsulinemic-euglycemic clamp) and total FM, visceral adipose tissue, and waist circumference. However, the authors found no association between adipose tissue characteristics and postprandial ∆RQ in response to dietary challenges. Nevertheless, the systematic review only included nine studies, and none had longitudinal measurements of body composition, which may have precluded the detection of relationships with postprandial ∆RQ measured in response to mixed-meal consumption.

Changes in ΔRQ in response to mixed-meal ingestion were also positively associated with flight-induced changes in FFM. Although no relationship between the changes in postprandial ∆RQ and any physical activity variables was noted, inflight total FFM was previously positively associated with overall physical activity, time spent in vigorous physical activity, and T2 relative workload in these same astronauts^13^. It is in line with previous data from our research group showing that habitual physical activity predicts variation in postprandial RQ on Earth^2,48^. In addition, while decreases in physical activity concomitant or not with simulated microgravity favor the decrease in the shift from fasting oxidation to carbohydrates use following meal ingestion, the performance of combined aerobic and resistive exercise training prevented, at least partially, bedrest-induced alterations in muscle outcomes known to participate in postprandial ∆RQ^15^, including decreases in mitochondrial oxidative capacity, whole-body oxidative capacity, and insulin sensitivity. Therefore, physical activity may modulate postprandial ∆RQ in space, similar to what was seen on Earth. These data collected on Earth and in space suggest that the relationship we observed between spaceflight-induced changes in FFM and postprandial ∆RQ may be partially mediated via the impact of the exercise countermeasure and/or overall physical activity on FFM. Because correlations cannot inform cause-and-effect relationships, we cannot rule out a reciprocal relationship. Some data point toward a potential role for altered RQ changes to metabolic challenge in the control of muscle mass^34^. Finally, the negative association previously observed in the ENERGY experiment between inflight overall exercise and inflight FM^13^ further suggests that physical activity/exercise may also affect the metabolic response to spaceflights via changes in FM.

To date, among the four bedrest studies during which resting energy expenditure was measured^6,7,49,50^, only one noted a decrease in resting metabolic rate, even after adjustment for changes in FFM^7^. In the present study, average resting energy expenditure both unadjusted and adjusted for FFM remained unchanged after several months in space. In contrast, while no change was observed in diet-induced thermogenesis during bedrest studies, post-breakfast energy expenditure was significantly greater inflight than on the ground. It is possible that food processing costs are greater in space than on the ground. This may also be due to the changes in macronutrient utilization. During the flight, the utilization of carbohydrates that have a thermic effect of 5–10% increased to the detriment of fat that has a thermic effect of food of 0–3% only^51,52^.

Strengths and limitations need to be acknowledged. This is the first study to investigate substrate oxidation in astronauts during 6-month missions onboard the ISS. We used a standardized mixed meal as a metabolic challenge to assess changes in postprandial RQ under physiological conditions, unlike insulin stimulation or an oral glucose tolerance test, which create supraphysiological conditions. However, the relatively high proportion of both fat and proteins in the test meal did not allow for testing postprandial ∆RQ to carbohydrate and assess its relationship with insulin sensitivity. Other limitations include the relatively small sample size due to the challenges inherent to inflight experiments, the fact that only male astronauts were included, and the use of averaged urinary nitrogen excretion measured in astronauts during previous flights. Although the assessment of the inflight diet’s macronutrient composition was reliable, assessing the astronauts’ habitual diet on the ground was challenging due to their hectic schedules. As a consequence, valid data were obtained for only seven astronauts. While precautions were implemented throughout the flight to control environmental and technical factors that could affect indirect calorimetry measurements, these factors may still have influenced the inflight values.

In conclusion, we showed that, as observed during bedrest studies, spaceflight is associated with a shift in substrate use in favor of carbohydrate oxidation. However, rather than being primarily caused by microgravity, it might be predominantly driven by the macronutrient composition of the inflight diet. Whereas no change was detected on average, the considerable between-astronaut variability in postprandial substrate use highlighted the influence of both body composition and inflight aerobic exercise on these metabolic outcomes. These data support the importance of maintaining stable FM and, hence, energy balance during spaceflight. Additionally, they support the role of physical activity in the regulation of postprandial substrate use in space and that, provided compliance is maintained, the exercise countermeasure as it is currently prescribed might prevent the emergence of disruptions in substrate use. Because substrate use affects metabolic health and probably other physiological systems, future research will need to optimize dietary and exercise treatments for the long-term space missions foreseen for planetary exploration.

## Methods

### Subjects

Eleven male astronauts from eight countries (United States, Japan, Italy, Canada, France, Germany, United Kingdom, and The Netherlands), who flew for six months onboard the ISS, voluntarily participated in the ENERGY experiment. None had a history of chronic disease, and all were healthy throughout the missions. The study was approved by NASA Institutional Review Board (IRB) under NASA 7116301606HR and by the European Space Agency (ESA) Medical Board and Japanese Space Agency (JAXA) Institutional Review Board for human experiments. The study was conducted in conformity with the policy statement regarding the use of human participants as outlined in the Declaration of Helsinki. Written informed consent was obtained from all astronauts.

### Overall study design

Each astronaut completed two strictly identical metabolic tests, one on Earth (ground) and one onboard the ISS (inflight). Both sessions were conducted under the supervision of ESA and French Space Agency (CNES) science officers and investigators from Toulouse Space Center (CADMOS, Toulouse, France). Before launch, a dry run of the experiments took place at the European Astronaut Center (EAC) in Cologne (Germany). On average, the ground session occurred 99 days (SD 78, median 66, IQR 38, range 45–293) before the flight at EAC. The astronauts were considered in energy balance with an average weight variation of −1.2% (SD 2.1, range −4.9–1.9) compared to a measurement obtained 146 days [SD 164] before. The flight session started before the last month onboard the ISS and after at least three months in space (108 days [SD 19, median 105, IQR 30, range 83–140] on average). The two sessions were separated by 158 days (SD 29, median 166, IQR 35, range 112–197). The 3–5-month inflight window was selected to provide spaceflight data while avoiding the preparation period for return to Earth.

To explore potential contributors to substrate oxidation changes induced by spaceflights, body composition was determined using isotopic dilution on the same day, and physical activity was estimated using a Sensewear Pro (SWP) multi-sensor activity monitor (Body media Inc®, Pittsburgh, PA, USA) worn for 10 days following the metabolic test day. Inflight exercise training during the two months preceding the research session was also obtained from the NASA exercise diary logs. Finally, the food quotient (FQ) was calculated from self-reported inflight food intake during the same 10-day period of physical activity measurement.

### Test meal challenge

**Fig. 4:**
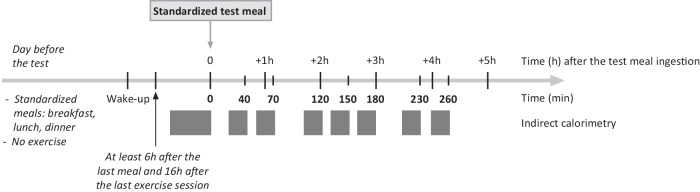
Schematic timeline of the test meal challenge protocol.

On the ground and inflight test days, energy expenditure and substrate oxidation were measured by indirect calorimetry in the fasting state and after a mixed-breakfast challenge. The measurements were performed after a minimal 6-hour overnight fast, at least 16 hours after the last exercise session, early after morning waking, after experimental device calibration (for the inflight session), and after resting for at least 45 minutes. The breakfast challenge was ingested in less than 20 minutes and designed to cover about one-third of the theoretical daily ground needs of the subjects, based on resting metabolic rate estimated from the Schofield equation using individual body weight, age, and sex, and an estimated physical activity level of 1.8. The energy content and macronutrient composition of the moderately high-fat and high-protein breakfast meal are presented in Table 3. Gas exchanges were measured for 45 minutes before breakfast (i.e., fasting state) and during seven 20-min periods that started 20, 50, 100, 160, 210, and 240 minutes after meal ingestion (T0). The protocol timeline is presented in Fig. 4.

**Table 3:** Test meal energy content and macronutrient composition

		Mean (SD)
Energy content	KJ.kg^−1^	54 (6)
	% ground needs^1^	34.6 (5.6)
Carbohydrates	g	93 (22)
	% energy	37 (9)
Lipids	g	38 (5)
	% energy	35 (4)
Proteins	g	67 (17)
	% energy	28 (8)

^1^Theoretical daily energy needs of the subjects on the ground, based on individual body weight and an estimated physical activity level of 1.8.

To minimize the confounding effect of recent diets, subjects were provided with standardized meals designed to cover subjects’ theoretical needs the day before the metabolic test. It represented a mean (SD) energy intake of 187 (25) kJ.kg^−1^.day^−1^ composed of 33% (4) carbohydrates, 38% (3) lipids, and 29% (3) proteins. About 30%, 40%, and 30% of the daily energy intake were given at breakfast, lunch, and dinner, respectively.

The breakfast-test meal and the standardized pre-test meals were prepared by French Chef Ducasse, which maximized astronaut compliance with the research protocol. After a preliminary tasting on the ground, meals were individually adapted to the subjects’ preferences and were identical for the ground and flight sessions. Dishes examples are presented in Supplemental Table 1.

### Indirect calorimetry

Throughout the test, O_2_ consumption (VO_2_) and CO_2_ production (VCO_2_) were measured by the pulmonary function system (PFS, manufactured by Danish Aerospace Company [DAC], Odense, DK]) validated for use in space^53,54^. Subjects were connected to the PFS by a custom-designed two-way non-rebreathing valve coupled to a mixing reservoir for expired gases. The PFS uses a photo-acoustic method for CO_2_ analysis (operating range from 0–12%) and an Oxigraf^TM^ model X2004 sensor (Oxigraf, Mountain View, CA), a laser diode absorption spectroscopy sensor, to measure O_2_ (operating range from 0–100% O_2_ with a resolution of 0.01%). A differential pressure flowmeter (pneumotach; range 0–900 L.min^−1^) is used for ventilation measurement on the inspired side of the non-rebreathing valve rather than on the expired side. Indeed, the decrease in contamination from moisture contained in expired gas usually relies on gravity, which obviously cannot work in space. Temperature, humidity, and inspired gas concentrations are measured on the inspired side of the respiratory circuit because inspired gas concentrations on the ISS slightly deviate from normal atmospheric values. A proprietary software package (DAC) was used to compute minute-by-minute VO_2_ and VCO_2_ and respiratory quotient (RQ as VCO_2_/VO_2_) from raw data signals measured by the PFS (VE, FeO_2_, and FeCO_2_) considering the delay time for the sensors to detect expired gas and the time shift to match the gas fraction data to ventilation. As inspired ventilation is measured, VO_2_ is calculated using the Haldane transformation. All data are reported under standard pressure and temperature conditions, i.e., 1.013 bar and 0 °C. Before each experimental session, the PFS underwent acceptance tests to validate instrument performance; the gas analyzer module was calibrated using reference gases, and the flowmeter was calibrated with a 3-L volumetric syringe. On the ground, a technician from the DAC calibrated the PFS and showed the participants how to proceed in space. To limit the effect on the measures of environmental conditions that differ on the ground and inflight, indirect calorimetry was performed in a dedicated room with controlled temperature at EAC. On the ISS, the PFS is located in the Human Research Facility Rack 2 of the Destiny Laboratory, which is isolated from the other living areas, and on the days of the tests, the other crewmembers were asked not to disturb the astronaut during the entire protocol.

### Energy expenditure and substrate oxidative rates

For each of the eight periods of indirect calorimetry measurement (one in fasting and seven in the postprandial state), the first 5 minutes were excluded, and the stability of the data was visually checked. VO_2_, VCO_2_, and RQ were then averaged over each of the eight time periods of gas exchange measurements. The amount of protein oxidized daily (normalized for body weight) was approximated from total urinary nitrogen excretion obtained in 130 astronauts in preflight conditions and 25 astronauts after 4 months on the ISS (Smith SM & Zwart SR, unpublished data), assuming that 1 g of nitrogen comes from ~6.25 g of protein. Averaged urinary nitrogen was 1.178 g.kg^−1^ on the ground and 1.195 g.kg^−1^ inflight. The Weir’s equation^55^ and Frayn’s equation^56^ were used to respectively calculate energy expenditure and the total oxidative rates of carbohydrates and fat.

The post-breakfast substrate oxidation response was expressed as 0-to-260 min iAUC calculated by the trapezoidal method. Fasting and post-breakfast substrate oxidations were expressed in both absolute values (grams per minute and grams over 260 min, respectively) and relative to the corresponding energy expenditure, i.e., fasting and post-breakfast energy expenditure iAUC respectively. The overall shift from fasting fat oxidation to carbohydrate oxidation following meal consumption was estimated from the difference between maximal post-breakfast RQ and fasting RQ, i.e. ΔRQ^57,58^. Inflight data from one subject was not included in the data analysis because his fasting RQ was >1.2, which is very unlikely and suggests a technical or other issue.

### Body mass and composition

Body mass was assessed on the first day of both sessions. A calibrated scale was used on the ground, and subjects were in undergarments. The SLAMMD (Space Linear Acceleration Mass Measurement Device) device was used inflight. Using the concept of linear acceleration, this device measures astronaut mass from 41 kg to 109 kg, with an accuracy of plus or minus 227 grams^59^. Before each use, the device is calibrated with a known mass. Once calibrated, the astronaut took five measurements, the three best of which were saved and averaged. FFM was obtained by isotopic dilution based on urine samples at equilibrium time from doubly labeled water (DLW) measurement as previously detailed^13,60^. Briefly, on the ground, the astronauts ingested 3.0 g.kg^−1^ total body water (TBW) of a pre-mixed dose of DLW providing 0.3 g.kg^−1^ TBW from 10% enriched H_2_^18^O and 0.15 g.kg^−1^ TBW from 99% enriched ^2^H_2_O. Inflight, the same DLW dose was used for all astronauts to simplify the inflight handling of materials. The dose was calculated to provide 0.45 g.kg^−1^ TBW of H_2_^18^O and 0.35 g.kg^−1^ TBW of ^2^H_2_O for an 80 kg astronaut^13^. FFM was calculated assuming a hydration coefficient of 73.2%^61^. FM was calculated as the difference between body mass and FFM. Body mass index (BMI), FFM index (FFMI), FM index (FMI) were calculated by dividing body mass, FFM, and FM (in kg) by squared height (in squared m).

### Physical activity and inflight physical training

The SWP, an activity multi-sensor armband worn on the non-dominant arm for the 10 days of ground and inflight sessions, was used to measure total exercise and non-exercise physical activity as previously detailed^13^. Its companion software (modified professional version 8.0) that incorporates a proprietary machine-learning activity classification algorithm based on heat flux, galvanic skin response, skin and near-body ambient temperature, and accelerometry measure patterns were used to identify non-wear periods and, after exclusion of non-valid days, minutes of total physical activity, walking and running. The norm of the 1-min acceleration-signal mean amplitude deviation (MAD)^62^ aggregated over activity periods was used as a proxy of physical activity workload (in milli-g, i.e., 0.001 g). MAD removes the static component due to gravity from the acceleration signal to only keep its dynamic component related to body movements; it is, therefore, poorly influenced by microgravity. Valid data were obtained for 11 astronauts on the ground and 9 astronauts inflight.

Inflight physical training sessions, prescribed six days a week, were composed of aerobic exercises performed on either a cycle ergometer (CEVIS) or a second-generation treadmill (T2) with a vibration isolation system and resistive exercises performed on the advanced resistive exercise device (ARED). Each astronaut’s inflight exercise regimen was compiled from the exercise diary logs that NASA provided. Inflight exercise parameters were calculated over a period beginning two months before the metabolic research session, as previously detailed^13^. Weekly resistance exercise repetitions and duration were both considered in this study.

### Food quotient

A 10-day food record completed with photos of meals, snacks, and drinks was used to assess macronutrient intake during the sessions on the ground and inflight. Inflight, it was associated with systematically scanning the barcode of nonperishable food portion packages provided on the ISS. Notes about the consumed quantity were also provided by the astronauts. This allowed a quite precise estimation of both the quantity and quality of the food consumed inflight for all the subjects. On the ground, exploitable food records for at least 6 days were obtained in 7 subjects. Corresponding energy and macronutrient contents were obtained from food tables provided by NASA, ESA, JAXA, the Russian Space Agency, and CADMOS for inflight data and from CIQUAL food table^63^ for ground data. Food quotient or FQ was calculated using the equations of Black^64^:

1 $${FQ}=\frac{\left(P.\times 0.91\times 0.781\right)+\left(F\times 0.965\times 1.427\right)+\left(C\times 0.746\right)+(A\times 0.973)}{\left(P\times 0.91\times 0.966\right)+\left(F\times 0.965\times 2.019\right)+\left(C.\times 0.746\right)+(A\times 1.461)}$$

with *P*, *F*, *C*, and *A* as protein, fat, carbohydrate, and alcohol intakes expressed as g.day^−1^.

### Statistical analysis

Linear mixed-effects models accounting for repeated measurements with subjects as random effects were used to test the impact of spaceflight on the anthropometric, activity, and metabolic variables. Heterogenous variance models were used in the case of variance inhomogeneity across ground and inflight periods. Additionally, FFM and FM were added as fixed effects in the models examining the impact of spaceflight on energy expenditure and absolute substrate oxidation rates, as these values directly depend on body mass and composition, which were individually impacted by spaceflight^13^. Models without adjustment on FFM and FM, which led to similar conclusions, are provided in Supplemental Table 2 for information. Our statistical inference was based on the net changes between ground and inflight in fasting and post-breakfast energy expenditure, substrate oxidation rates, and RQ with their 95% confidence interval (CI).

Similar linear mixed-effects models accounting for repeated measurements were used to examine the associations of specific individual metabolic data (e.g., fasting and post-breakfast RQ and substrate oxidation variables) measured both on ground and inflight, as dependent outcomes with corresponding FQ values as explanatory variables. Multilevel mediation analysis was used to examine whether the impact of spaceflight on RQ was mediated by FQ^65^.

Then, general linear models were used to examine the associations between changes (difference between inflight and ground values) in RQ and in fasting- and post-breakfast substrate oxidation as dependent outcomes with successively (1) changes in body mass, FFM, FM, and physical activity, and (2) inflight exercise training (of note, no data were available for astronaut’s exercise training on the ground). Finally, the association between changes in RQ and inflight FQ, controlling for ground values of RQ (to indirectly account for ground FQ assuming that astronauts were in energy balance before the flight) was examined.

Descriptive anthropometric, physical activity, and diet-related values are presented as means (standard deviation, SD). Unless otherwise noted, results of the linear mixed-effects models examining the impact of spaceflight on the different outcomes are presented as least square means (LSmeans) with their standard error (SE) or 95% confidence interval (95% CI). LSmeans estimated from models adjusting for FFM and FM were calculated using whole group mean ground FFM and FM values (63.77 and 15.59 kg, respectively). Statistical analyses were performed using SAS 9.4 (SAS Institute, Cary, North Carolina) with a significance level of 5%. Figures were realized with Prism 9 (GraphPad, San Diego, California).

### Reporting summary

Further information on research design is available in the Nature Research Reporting Summary linked to this article.

### Supplementary information

Supplemental material

Reporting Summary

## Data Availability

Data are accessible upon request to the authors.
