# Supplementary material for: Substrate metabolism in male astronauts onboard the International Space Station: the ENERGY study
Source: NPJ Microgravity. 2024 Mar 27;10:39. doi: 10.1038/s41526-024-00360-0 (PMC10973451; doi:10.1038/s41526-024-00360-0)
Supplement: Supplementary file 1 — Supplemental material [file 41526_2024_360_MOESM1_ESM.pdf]

Substrate metabolism in male astronauts onboard the International Space Station: The ENERGY study

Elisa Le Roux[1], Alexandre Zahariev[1], Isabelle Chery[1], Dale A. Schoeller[2], Pierre Bourdier[1], Alain Maillet[3,4], Cecile Thevenot[3,4], Maël Garnotel[1], Guillemette Gauquelin-Koch[5], Laurie Van Den Berghe[6], Stéphane Blanc[1$], Chantal Simon[6,7$] and Audrey Bergouignan[1,8$]

Affiliations:

[1]CNRS, IPHC UMR 7178, University of Strasbourg, Strasbourg, France

[2]Department of Nutritional Sciences, University of Wisconsin, Madison, USA

[3]MEDES, Institut de Médecine et Physiologie Spatiale, Toulouse, France

[4]CADMOS-CNES, Toulouse, France

[5]Centre National d'Etudes Spatiales, Paris, France

[6]Human Nutrition Research Centre of Rhône-Alpes, Hospices Civils de Lyon, Lyon, France

[7]CarMen Laboratory, INSERM 1060, INRA 1397, University of Lyon, Oullins, France

[8]Division of Endocrinology, Metabolism and Diabetes, Anschutz Health & Wellness Center, Anschutz Medical Campus, University of Colorado, Aurora, CO, USA

[$]co-last authors

Corresponding author:

Audrey Bergouignan

CNRS Institut Pluridisciplinaire Hubert Curien UMR7178

Département d'Ecologie, Physiologie et Ethologie

23 rue Becquerel, 67087 Strasbourg Cedex, France

Tel: +33388106914

Email: audrey.bergouignan@iphc.cnrs.fr

Running title: Nutrient metabolism during spaceflights.

Supplemental Table 1: Examples of dishes proposed to astronauts for standardized meals.

Recipes	Nutritional values kcal per 100g)	Macronutrients composition		
		Proteins	Lipids	Carbohydrates
**Salmon with candied Menton lemon**	246	35.0	11.7	0.2
**Rivieira style swordfish**	191	12.0	11.0	0.0
**Britany lobster, quinori with seaweed, Menton lemon condiment**	194	12.9	5.7	22.8
**Scottish salmon, candied tomatoes and grilled eggplant**	281	33.2	15.5	2.2
**Shredded chicken Parmentier**	266	26.6	13.1	10.6
**Spiced chicken stir-fried thaï vegetables**	276	56.2	4.6	0.0
**Duck confit with capers**	396	46.5	23.3	0.2
**Lamb shoulder confit with sage, pearl barley and candied tomatoes**	269	21.7	11.0	22.0
**Landes yellow poultry cooked as "Poule au pot"**	280	36.4	5.0	12.6
**Beef cheeks in Bourguignon style, carrots and mushrooms**	197	29.9	7.26	2.64
**Carrots tops**	29	0.6	0.4	6.5
**Caponata**	62	1.9	3.2	9.5
**Vegetables and tomatoes fondue**	36	0.9	0.9	6.8
**Omelet cake with tomatoes and herbs**	125	11.9	5.9	6.7
**Egg cocotte, Basque style condiment**	112	6.4	6.7	7.4
**Cheese-cake**	191	4.0	12.5	15.6
**Muesli**	282	5.5	14.3	33.9
**Chocolate cake**	340	5.0	27.9	18.8
**Semolina with dried apricots**	269	9.0	2.3	52.0
**Apple fondant pieces**	74	0.2	0.5	18.1
**Creamy lemon baked**	250	19.1	19.1	17.4

Supplemental Table 2: Ground and inflight energy expenditure and substrate oxidation data of the 11 astronauts (models non adjusted for FFM and FM)

	Ground	Inflight	Changes from ground[1]	
	**LSmeans (SE)**	**LSmeans (SE)**	**LSmeans (95%CI)**	**P Value**
Fasting state[2]				
**Energy expenditure (KJ/min)**	4.73 (0.23)	4.52 (0.24)	-0.22 (-0.54 to 0.11)	0.17
**Glucose oxidation (g/min)**	0.13 (0.02)	0.18 (0.02)	0.06 (0.01 to 0.11)	0.03
**Lipid oxidation (g/min)**	0.04 (0.01)	0.01 (0.00)	-0.03 (-0.05 to -0.01)	<0.01
Post breakfast-challenge (iAUC over 260 min)[2]				
**Energy expenditure iAUC (kJ)**	302 (31)	447 (55)	145 (9 to 280)	0.04
**Glucose oxidation iAUC (g)**	16.81 (6.13)	21.70 (3.74)	4.89 (-10.30 to 20.09)	0.51
**Lipid oxidation iAUC (g)**	0.95 (2.23)	2.91 (0.80)	1.97 (-3.17 to 7.10)	0.42

[1]Estimated LSmeans (95%CI) and P value from mixed-effects models accounting for repeated values.

[2]inflight data from 10 subjects.

iAUC, incremental area under the curve over the 260 min period of measurement

Supplemental Table 3: Multiple regression analysis predicting changes in fasting RQ in the astronauts (data for 10 astronauts).

Model Summary

Global R-square	RMSE
**0.90**	**0.024**

ANOVA

Source	df	Sum of Squares	Mean square	F value	P-value
**Model**	**2**	0.03460356	0.01730178	30.03	0.0004
**Error**	**7**	0.00403339	0.00057620		
**Total**	**9**	0.03863695			

Parameters

Predictors	Estimate (SE)	t-value	P-value
**(Intercept)**	-1.35 (0.50)	-2.69	0.03
**Inflight food quotient**	2.41 (0.56)	4.28	<0.01
**Ground fasting RQ**	-0.78 (0.12)	-6.24	<0.001

RQ, respiratory quotient.

Partial R square for inflight food quotient: 0.72 (P=0.002)
